# Microcapsules in Fiber Metal Laminates for Self-Healing at the Interface between Magnesium and Carbon Fiber-Reinforced Epoxy

**DOI:** 10.3390/ma16196524

**Published:** 2023-10-01

**Authors:** Monika Ostapiuk

**Affiliations:** Department of Materials Engineering, Faculty of Mechanical Engineering, Lublin University of Technology, 36 Nadbystrzycka St., 20-618 Lublin, Poland; m.ostapiuk@pollub.pl

**Keywords:** FML, microcapsules, interface, SEM

## Abstract

The paper presents issues regarding self-healing isophorone diisocyanate (IPDI) microcapsules applied in fiber metal laminates (FMLs) based on magnesium AZ31 and a carbon-reinforced polymer composite. The aim of this work is to analyze the morphology of the produced PU/PUa microcapsules containing IPDI and their behavior in a self-healing layer in FMLs. By means of SEM, it was confirmed that the microcapsules (MCs) had a narrow size distribution. Therefore, the developed MCs, having sizes ranging from 10 to 35 µm, might contribute to an improved self-healing coating, which benefits from the advantages of both small and larger-sized MCs. The positive result of the work is that there is a possibility to improve the properties of FMLs with added MCs, i.e., in corrosion and mechanical phenomena.

## 1. Introduction

In response to the demand for new materials, there has been an increased number of scientific publications in the field of materials engineering and biomedical science regarding the development and improvement of new magnesium alloys aimed to be used in various fields. The advantages of magnesium alloys include, among others, a low density and an environmentally safe composition. Their tendency to suffer from corrosion imposes a serious difficulty. A plasma electrolytic oxidation (PEO) layer, which is the most popular method for the surface treatment of magnesium alloys in fiber metal laminates (FMLs), requires additional reinforcement in the form of various types of primers to increase adhesion. In one work, the authors presented an FML with additional protection against corrosion and with the capacity for self-healing via the addition of MCs [1]. MCs play a double role in anti-corrosion coatings, inhibiting both the spread of corrosion and cracks in a structure, avoiding the penetration of aggressive substances into the metal when the coating is damaged. FMLs are quite attractive, promising materials when it comes to the use of a magnesium alloy, e.g., AZ31 combined with a carbon/epoxy polymer composite. In addition, the use of MCs as compounds capable of polymerization and which are sensitive to external stimuli has been recognized as a viable, attractive, and intelligent way to introduce therapeutic functions to materials [2,3].

The incorporation of MCs in FMLs is a new idea in the industry, with there being several components that can be used to build and fill MCs. Various solutions have been designed and tested in terms of active substances and functionalized coating matrices to achieve good compatibility between MCs and coatings.

When the self-healing of a structure is needed, four conditions should be met: storage, release, transport, and rebonding. For this application, all the conditions are dependent on the chemical composition and properties of the self-healing agent. The MCs should provide enough protection so that the healing agent inside the MCs remains stable for a long period of time (until it is released into the damaged zones by cracking) [4,5].

The shells of MCs are broken when mechanical rupture occurs, releasing the healing agent. For this application, it is required that the MC shells be able to withstand the coating process and retain their encapsulated contents until mechanical damage is inflicted, as a result of which it is expected that the shells will break, releasing the encapsulated contents. The authors, Ostapiuk et al., demonstrated that the produced polyurethane/polyurea (Pu/PUa) MCs were able to endure the specific pressure needed to polymerize a layer of a carbon/epoxy composite during FML production [6]. White et al. [7] are credited with being the first to develop self-healing polymer composites, in which the monomeric healing agent, dicyclopentadiene (DCPD), was encapsulated and dispersed in an epoxy matrix along with bis(tricyclohexylphosphine) benzylidine ruthenium (IV) dichloride (Grubbs catalyst) for a catalytic chemical trigger [5].

In their groundbreaking work, White et al. conducted crack experiments in which as much as 75% of the original crack resistance was recovered in clean samples of epoxy resin after 48 h of recovery time [8]. This approach was further refined by adjusting the size of the microcapsules and the concentration of the catalyst in [8] to produce a self-healing polymer that could recover up to 90% of its original fracture toughness [5].

**Figure 1:**
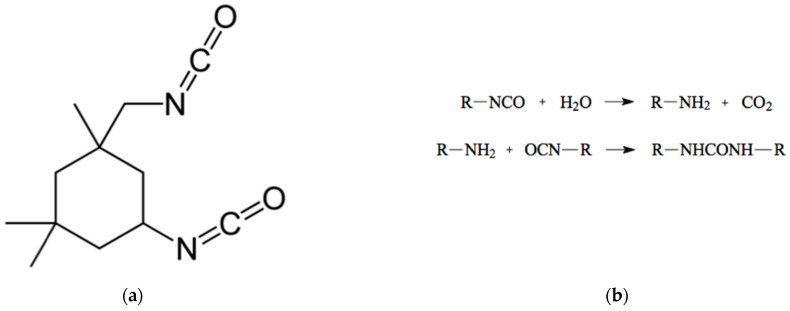
(**a**) Isophorone diisocyanate (IPDI) and (**b**) reaction of IPDI with water.

When it comes to damage repair, polymerization kinetics are quite an important parameter. However, the ability of MCs to break by means of mechanical damage and self-heal a coating depends not only on the healing kinetics, but also on the applied forces and the duration of action on the laminate structure. A highly reactive but very attractive healing agent is isophorone diisocyanate (IPDI), as a one-component and catalyst-free agent [7,9]. After being released from MCs, IPDI effectively reacts with water/moisture (necessary for the development of corrosion) and forms a solid material that protects the metallic substrate against corrosion. The chemical reaction of IPDI with water is shown in Figure 1.

Successful self-healing in response to external mechanical stress mainly depends on (i) the properties of the active ingredient, (ii) the diameter of the MCs, and (iii) the properties of the MC shell. The MC shell material should be (i) chemically stable in the coating formulation, (ii) rigid enough to maintain integrity during mixing and application, and (iii) brittle enough to break if mechanically damaged [5,8]. The latest reports in the literature show that the best results are obtained by using isocyanate compounds as self-healing agents, focusing mainly on the encapsulation of methylene diphenyl diisocyanate (MDI), toluene diisocyanate (TDI), hexamethylene diisocyanate (HDI), and isophorone diisocyanate (IPDI) [9,10].

The aim of this work is to analyze the morphology of the produced PU/PUa microcapsules containing IPDI and their behavior at the interface in FMLs. Compared to other articles by the present author [1,6], this article gives a new, closer look at the morphology of the MCs. In addition, EDS is performed on the main elements that make up the MCs. The morphology and EDS of the layer with the microcapsules are presented. The interface between the Mg layer with PEO and the resin containing MCs is investigated. In this article, a basic analysis of the locations of MCs on the interface is provided. The need for such a presentation results from the lack of observations of FMLs with MCs located on the interface. The results could help us to understand the structures of MCs at the interface, to provide the next steps for research.

## 2. Materials and Methods

Isophorone diisocyanate (IPDI, with 98% purity) with the commercial name of Desmodur® I was encapsulated. It was obtained from Covestro AG (Leverkusen, Germany). A polyisocyanate of the commercial name Desmodur^®^ RC, used as the shell precursor, was kindly supplied by CIPADE S.A (S. João da Madeira, Portugal). The emulsion stabilizer gum arabic (GA) was obtained from Fisher Chemical (Porto, Salvo, Portugal). A poly(ethyleneimine) (PEI) aqueous solution (Mw 60,000, 50 wt% in H_2_O) and triethoxy(octyl)silane (n-OTES) were used. They were obtained from Sigma-Aldrich (St. Louis, MO, USA). Desmodur RC is much more reactive than Desmodur I; therefore, the former quickly and preferentially reacts at the interface, forming the shell.

### 2.1. Microcapsule Preparation

Interfacial polymerization processing in combination with an oil-in-water (O/W) microemulsion system was used for the MC production. The emulsion was prepared by vigorous stirring of the O and W phases, the first consisting of 4.85 wt% of the emulsion, at the speed of 1200 rpm, for 10 min at room temperature (RT), using an Ultra-Turrax Crushing Disperser (IKA T25 digital ULTRA TURRAX, Algés, Germany). The organic phase of the emulsion was composed of two isocyanates, Desmodur^®^I, the one to be encapsulated, and Desmodur^®^ RC, which was a mixture of an isocyanate prepolymer in ethyl acetate at 25 wt%. The first isocyanate, the one to be encapsulated, comprised 51.2 wt% of the O phase, and Desmodur^®^ RC comprised the remaining 48.8 wt%. The W phase was composed of water and gum arabic (GA) as the emulsion stabilizer, present at 4.7 wt%. The emulsion system was subjected to mechanical stirring at 500 rpm, at 50 °C, and the active H sources n-OTES (triethoxy(octyl)silane) and an aqueous solution of PEI (polyethylenimine) were added to contribute to the MC shell formation. The synthesis was maintained under the abovementioned conditions for 3 h 30 min. The completion of MC synthesis was confirmed by the evaluation of samples taken during the synthesis, by light microscopy. When the MCs were ready, they were washed with water to avoid aggregation. The final MCs were dried at atmospheric pressure and ambient temperature for 96 h before being stored.

### 2.2. FML Preparation

The FML was based on the magnesium AZ31 alloy (0.5 mm, Luoyang Shengte Corp., Henan, China) and unidirectional carbon fiber/epoxy prepreg tape (0.131 mm for one layer, AS7J Hexcel, Stanford, CA, USA). The FML configuration was 2/1. The surface of the AZ31 magnesium alloy was specially prepared by the PEO process carried out in several steps, which were the following. The surface was cleaned/degreased in an alkaline Na_2_SiO_3_ solution and activated in 10% HF with rinsing in distilled water. The aqueous electrolyte used for the PEO process contained 40 g/L sodium hydroxide and 50 g/L sodium metasilicate Na_2_SiO_3_. A high voltage of up to 400 V and a DC power source were used to supply a unipolar pulse of 0.5 ms and 4.5 ms, “on” and “off” pulsed time. A constant current (5 A/dm^2^) was used. This corresponded to the work cycle and frequency of 200 Hz. The PEO treatment was conducted for 10 min. The coated samples were cleaned in distilled water and then dried in controlled ambient-like conditions (Micro Arc, Gliwice, Poland). The pH was 13.1.

The AZ31 sheets with the PEO layer were coated with a sol–gel layer. The commercial EC 2333 (3 M Scotch WeldTM, St. Paul, Cottage Grove, MN, USA) primer, based on resin and inorganic–organic silanes (sol–gel based on toluene, 3-(trimethoxysilyl)propyl glycidyl ether), was used. The employed primer was applied by means of an atomizer on the Mg/PEO surface after being mixed with selected amounts of MCs. Afterwards, the samples were dried for 72 h at RT. The FMLs were manufactured in an autoclave chamber with pressure of −0.08 MPa, a curing temperature of 135 °C, a curing time of 2 h, and heating/cooling of 0.033 K/s. The vacuum in the autoclave was −0.02 MPa and it was applied throughout the cure cycle.

**Figure 2:**
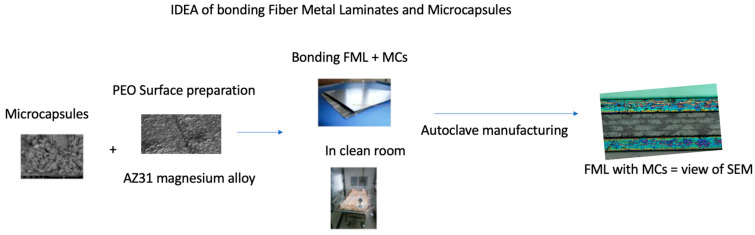
Scheme of FML manufacturing.

In Figure 2, the scheme of FML manufacturing is shown according to the steps involved in preparing the whole structure.

### 2.3. SEM Analysis

A high-resolution scanning electron microscope, the Nova Nano SEM 450 (FEI, Eindhoven, The Netherlands), was used for the morphological and microstructural analysis. Secondary electron (SE) imaging under 5.0 and 30.00 kV accelerating voltage conditions was used. A low vacuum pressure of approximately 90–120 Pa was applied in SEM. The Image Pro Plus program was used to count and number the MCs.

## 3. Results

### 3.1. Morphology of Microcapsules and PEO Layer

**Figure 3:**
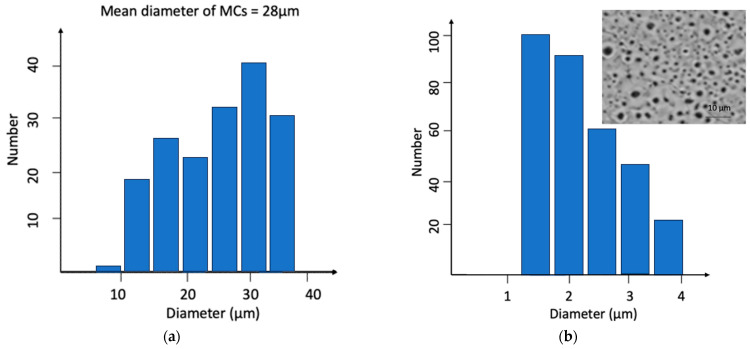
Size distribution of (**a**) microcapsules and (**b**) craters in PEO layer.

Figure 3a presents a graph of the particle size distribution per volume of MCs filled with IPDI. MCs with diameters between 10 µm and 35 µm were obtained, with the average value of 28 µm, which can be considered a relatively wide size distribution for the current application [11]. The size distribution of the craters in the PEO layer is shown in Figure 3b.

Most of the craters in the PEO layer were approximately 1.5 µm; however, there were some larger craters with diameters not exceeding 3.7 µm, which were present in the lowest number. The MC size distribution might broaden as a result of increasing the shear forces acting on the emulsion droplets during the initial phase of MC formation [10,11]. It was shown that in the case of self-healing coatings, the amount of self-healing agent available to fill the crack area depends on the MC shell thickness and MC size, with larger MCs having a higher ratio of core content per surface area [10,11].

**Figure 4:**
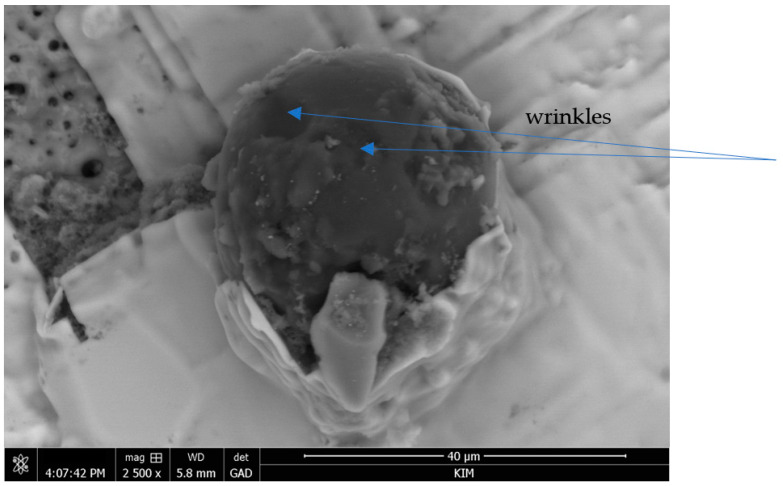
View of MC in sol–gel matrix; SEM.

**Figure 5:**
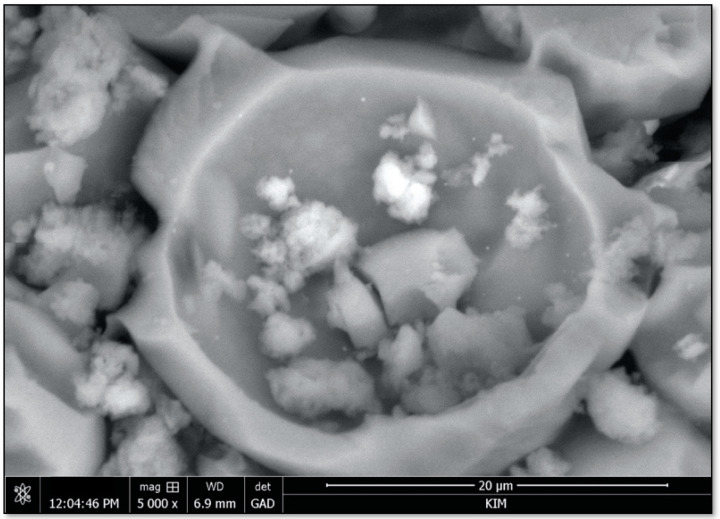
View of broken MC.

All the obtained MCs had a wrinkled surface (Figure 4), which is in line with the results reported in the literature for PU microcapsules filled with IPDI [12,13,14]. This is usually attributed to fluid-induced shear forces caused by the mechanical stirring employed during the synthesis, adding to the inhomogeneous formation of the shell as a result of the different reaction kinetics of the isocyanate with the active H sources and water [13,15,16]. It is also known that slow shell formation leads to an initially thin and flexible shell, which tends to wrinkle due to a reduction in the MC size caused by the progress of interfacial polymerization reactions [13,15,16]. These wrinkles may, among other consequences, increase the mechanical interaction between the microcapsule shell and the polymer matrix as they create an increase in the available surface area. The rough and wrinkled surface also depends on the properties of the core material (i.e., the solvent content, volatile component condensation, water solubility, core to shell ratio and the encapsulation procedure [14]). The microphotography of the wall thickness of the synthesized microcapsule is shown in Figure 5.

It can be seen that the MCs have a core–shell morphology, and the average shell thickness is approximately 2.4 µm. The inside of the MCs is distinctly smooth and the shell has no internal porosity

**Figure 6:**
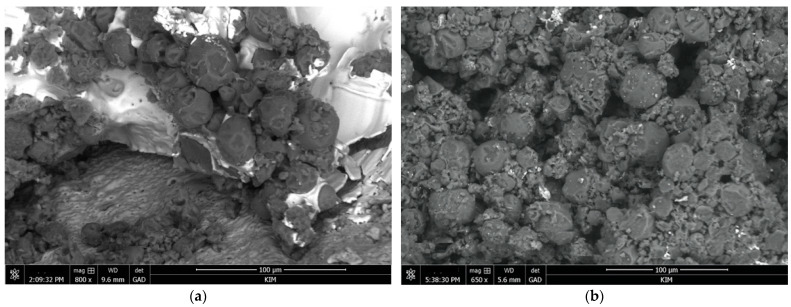
MCs visible on PEO layer: (**a**) with visible sol–gel and (**b**) accumulation on PEO layer.

SEM micrographs of the surface of the PEO coating with various amounts of MCs are shown in Figure 6.

**Figure 7:**
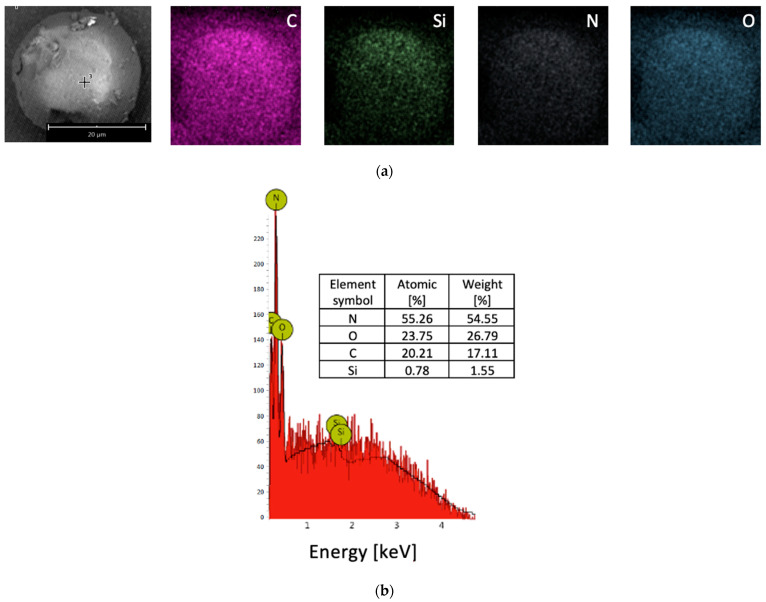
EDS with (**a**) mapping and (**b**) chemical elements in MCs.

The SEM micrographs and EDS identification of the elements of the MCs are shown in Figure 7. The elements from the MCs were recognized as C, Si, N and O.

In self-healing systems, the healing efficiency strongly depends on the core content, type and size of MCs. In general, increasing the concentration and size of the MCs improves the healing properties of the coating, owing to more available core material to be released. However, because of the thickness, appearance and mechanical properties of the coating layer, it is undesirable to use a very high concentration of large MCs. In order to achieve a balance between the self-healing properties of the coating and its mechanical properties, the size and concentration of the MCs embedded in the coating composition must be optimized.

The variability of the core to shell ratio strongly affects the stiffness of the MCs, as well as the healing efficiency and mechanical properties of samples with coatings containing these MCs. By increasing the ratio of the core to the shell, the amount of available healing agent in the MCs grows and, in turn, the repair quality of the damaged areas and the mechanical properties of the repaired coating, delaying corrosion phenomenon. Various types of primers are usually used due to their attractive feature of adhesion to the substrate. In the case of testing a magnesium alloy AZ31 PEO layer with MCs, a sol–gel layer was additionally added.

### 3.2. FML with MCs

**Figure 8:**
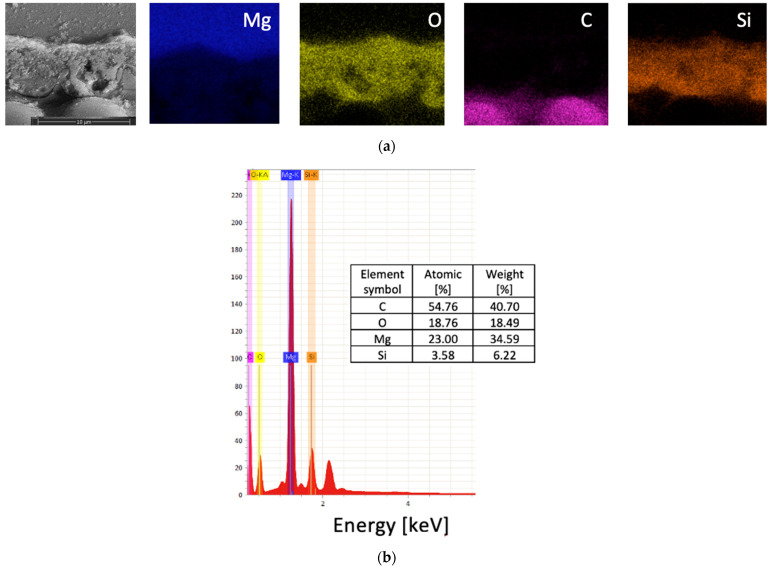
EDS with (**a**) mapping and (**b**) chemical elements in FML.

The SEM micrographs of the FML cross-section corresponding to the EDS are presented in Figure 8. The EDS mapping analysis showed that FMLs based on PEO coatings had Mg and O, while Si was also detected. Additionally, it was shown that C corresponded to the carbon fibers.

**Figure 9:**
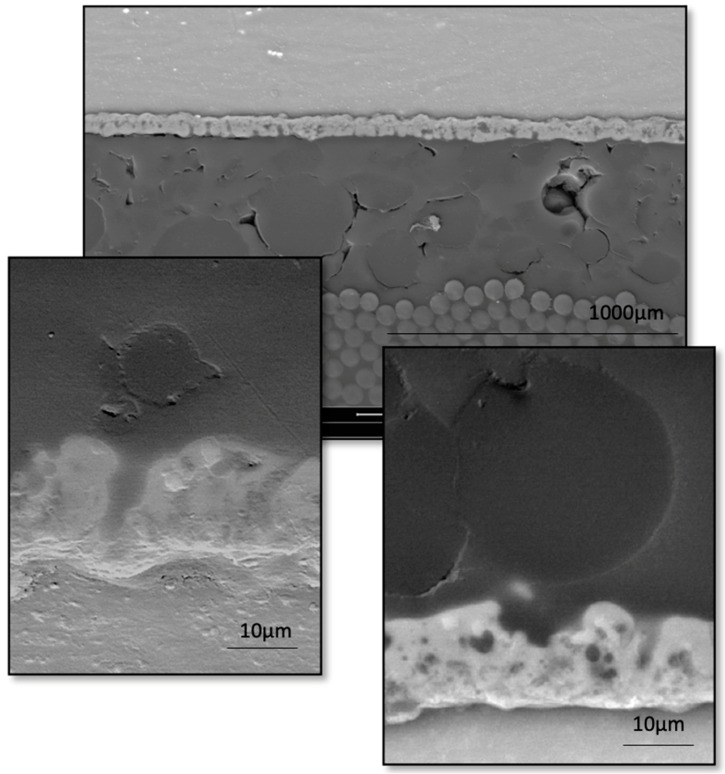
Microstructure of FML laminate with MCs and visible MgO layer.

In Figure 9, an SEM micrograph of an FML laminate with MCs is shown.

The layer is porous, which is presented in more detail in the article by Ostapiuk [6]. Next, the layer containing the MCs and the carbon/epoxy composite is shown, in which it is possible to observe the good dispersion of the MCs. This will then be responsible for good self-healing and a balanced structure. The fibers are placed parallel to the layer, which is responsible for the arrangement of the fibers in the 0° direction. The dominant role in achieving the appropriate tensile strength and modulus of elasticity in the longitudinal direction (fiber arrangement) is played by the composite, while the metal layers affect the properties in the transverse direction. As a result, the tensile strength of unidirectional FMLs is significantly higher compared to the metal material used. On the other hand, the properties in the transverse direction for unidirectional laminates are slightly lower than those of the metal material. The use of a cross arrangement of composite layers allows the same strength values both in the longitudinal and transverse directions to be achieved. In Ostapiuk et al. [1], the research focused on FMLs with different fiber directions. The mechanism of self-healing is based on the compound inside the MCs. The visible layer of MCs is of equal thickness, which is a very good phenomenon and accordingly it will be able to self-heal. It is possible to observe MCs of various sizes, all of them in the desired size range. The interface of the MCs with the PEO coating on the AZ31 alloy shows the large size of the MCs compared to the size of the craters/porosity of the coating. The MCs are not small enough to enter the craters, which should be considered in future research into the fabrication of such MCs. They will then be able to improve the adhesion at the interface.

## 4. Discussion

The growing requirements for construction materials used in technology impose the development of advanced materials that allow the expected mechanical properties, better construction efficiency and durability to be obtained. Over the last few decades, polymer matrix composites reinforced with continuous fibers have become one of the basic and most promising groups of materials, especially in aerospace applications. This is related to the achievable significant modification of the mechanical and physical characteristics under the influence of introducing reinforcing fibers to the matrix, which makes composites extremely interesting both in terms of knowledge and application. Some of the most important materials connecting metal and composite continuous fibers are FMLs. The most important issue in FMLs is the formation of the metal/composite interface, related to the proper method of preparing the metal surface. Achieving specific physicochemical properties (e.g., topography and surface morphology) is necessary because they have a decisive impact on obtaining an adhesive bond with high strength and durability of the structure. An effective method of surface preparation allows the production of a high-quality, repeatable surface, capable of transferring high stresses and resistant to environmental conditions. On the other hand, the lack of a good adhesive bond between the metal and the fibers in the composite may lead to unfavorable phenomena on the interface—delamination. The analysis of destruction processes requires not only good knowledge of the factors that cause them and the conditions in which a given material can be destroyed, but also thorough knowledge of their mechanisms. The variety of destruction processes makes it of technical and economic importance and it is the subject of intensive scientific research. In FMLs, crack growth is inhibited due to the presence of the composite as a result of the bridging effect, which consists in transferring stresses from the damaged metal to the intact reinforcing fibers in the composite. A propagating crack in the metal layer prevents it from transferring tensile loads at the point of discontinuity. Nevertheless, through the metal/composite adhesive bond, the stresses are transferred from the damaged metal layer to the intact composite layer.

MCs could function to support the formed delamination or debonding in FMLs. This article characterizes MCs that have been specifically developed for FMLs. Isocyanate is envisaged to increase the adhesion strength in FMLs. This was discussed by Loureiro M. et al. [16] in research where the adhesive joint underwent a cross-linking phenomenon with isocyanate in a peel test. The strength of adhesion is not shown in this article, but it seems that the bond is strong at the interface between the MCs and AZ31. This was demonstrated by Ostapiuk et al. in [1] based on the ILSS test with the phenomena of self-healing. Shaping the properties of the adhesive connection in FMLs is a very complex issue. The main factors affecting the adhesive properties at the metal/composite interface include the structure, the chemical and phase composition of the interface, the topography and morphology of the surface layer, structural defects, the energy state of the metal surface and the properties of the bonding substrate. The properties of FMLs, apart from the selection of individual components, are conditioned, among others, by the characteristics of the metal/composite adhesive connection. Obtaining high strength and durability in the connection (adhesion) of the composite material to the metal substrate depends mainly on the preparation of the surface of the base metal. A lack of sufficient adhesion affects the possibility of structural discontinuities in the form of porosity and delamination, directly affecting the quality of laminates. Surface preparation processes should contribute to obtaining the maximum joint strength and repeatable joints and ensuring their long service life. In this case, the longer use of materials translates into a longer service life for the elements. Smart healing coatings typically perform the main healing function of repairing the polymer network via the presence of polymerizing agents and other materials, a good example of which is an FML composite. 

Additionally, self-healing coating technology is recommended for the corrosion protection of metal surfaces, in which the coating is exposed to physical damage due to impact and corrosion changes. In the case of using microcapsules (MCs) as a self-healing agent, adding to the inhibition of corrosion, MCs also promote the self-healing of the coating during mechanical damage. The MCs used for self-healing applications have the ability to release corrosion inhibitors in a controlled manner and can be used to develop a new family of smart multifunctional materials. The corrosion of metal structures causes significant financial losses. To overcome this, it is of major importance to find suitable anti-corrosion agents and to choose effective and economical corrosion protection technologies. The problem here is that magnesium alloy-based FMLs may be part of the component. In the next article, the self-healing phenomena of corrosion resistance in FMLs with MCs will be shown. There is currently a research focus on new types of anti-corrosion coatings that can intelligently respond to damage and automatically restore their functionality, preventing the corrosion of metal substrates.

Coatings for anti-corrosion protection can be divided into metallic, inorganic and organic. Organic coatings are widely used in industry, where, e.g., polymer coating systems are applied to the surfaces of metals to provide a dense barrier against corrosive substances. Nonetheless, organic anti-corrosion coatings undergo changes in mechanical, chemical or physical properties during their life, leading to the formation of micro-cracks, which propagate, exposing the substrate to moisture and oxygen. They lead to the accelerated separation of the coating at the metal/coating interface and its corrosion. Coatings cannot withstand corrosive agents such as water, oxygen and ions for long periods of time, meaning maintained periods, contributing to the tendency for the formation of micro-cracks during transportation and servicing, which is a huge hidden threat to metal substrates and has a direct impact on industrial facilities, the environment and human safety. Hence, the development of new solutions to avoid the occurrence of corrosion and to prolong the product shelf-life is of major importance, by means of decreasing the tendency for the internal cracking of anti-corrosion coatings; it is also important to improve their adhesion to the substrate.

## 5. Conclusions

In this article, MCs as part of an additional layer between CFRP and AZ31 in FMLs were investigated. By using the interfacial polymerization technique in combination with an O/W emulsion system, it was possible to produce PU/PUa core–shell MCs to be used as self-healing agents for Mg alloys. It was confirmed by SEM that the MCs had a narrow size distribution with an average size of 28 µm. As small-sized MCs are prone to having lower encapsulation content, the addition of larger amounts of MCs to guarantee the necessary core material for self-healing is required. On the other hand, the significant addition of larger-sized MCs also has an impact on the coating. Thus, the currently developed MCs, having sizes ranging from 10 to 35 µm, might contribute to an improved self-healing coating, benefiting from the advantages of both small and larger-sized MCs. The epoxy bonds the MCs and the AZ31 mg alloy in the interface. 

## Data Availability

The data presented in this study are available on request from the corresponding author.
